# Impact of Hypoxia-Ischemia on Neurogenesis and Structural and Functional Outcomes in a Mild–Moderate Neonatal Hypoxia-Ischemia Brain Injury Model

**DOI:** 10.3390/life12081164

**Published:** 2022-07-30

**Authors:** Anne Ehlting, Margit Zweyer, Elke Maes, Yvonne Schleehuber, Hardik Doshi, Hemmen Sabir, Maria Eugenia Bernis

**Affiliations:** 1 Department of Neonatology and Pediatric Intensive Care, Children’s Hospital, University of Bonn, 53127 Bonn, Germany; anne.ehlting@web.de (A.E.); margit.zweyer@dzne.de (M.Z.); elke.maes@dzne.de (E.M.); yvonne.schleehuber@dzne.de (Y.S.); maria.bernis@dzne.de (M.E.B.); 2 Deutsches Zentrum für Neurodegenerative Erkrankungen (DZNE), 53127 Bonn, Germany; hardik.doshi@dzne.de

**Keywords:** hypoxia-ischemia, neonatal, mild HIE, neurogenesis, behavior testing, MRI, BrdU, hypothermia

## Abstract

Hypoxic-ischemic encephalopathy (HIE) is a common type of brain injury caused by a lack of oxygen and blood flow to the brain during the perinatal period. The incidence of HIE is approximately 2–3 cases per 1000 live births in high-income settings; while in low- and middle-income countries, the incidence is 3–10-fold higher. Therapeutic hypothermia (TH) is the current standard treatment for neonates affected by moderate–severe HIE. However, more than 50% of all infants with suspected HIE have mild encephalopathy, and these infants are not treated with TH because of their lower risk of adverse outcomes. Despite this, several analyses of pooled data provide increasing evidence that infants who initially have mild encephalopathy may present signs of more significant brain injury later in life. The purpose of this study was to expand our knowledge about the effect of mild–moderate hypoxia-ischemia (HI) at the cellular, structural, and functional levels. An established rat model of mild–moderate HI was used, where postnatal day (P) 7 rats were exposed to unilateral permanent occlusion of the left carotid artery and 90 min of 8% hypoxia, followed by TH or normothermia (NT) treatment. The extent of injury was assessed using histology (P14 and P42) and MRI (P11 and P32), as well as with short-term and long-term behavioral tests. Neurogenesis was assessed by BrdU staining. We showed that mild–moderate HI leads to a progressive loss of brain tissue, pathological changes in MRI scans, as well as an impairment of long-term motor function. At P14, the median area loss assessed by histology for HI animals was 20% (*p* < 0.05), corresponding to mild–moderate brain injury, increasing to 55% (*p* < 0.05) at P42. The data assessed by MRI corroborated our results. HI led to a decrease in neurogenesis, especially in the hippocampus and the lateral ventricle at early time points, with a delayed partial recovery. TH was not neuroprotective at early time points following mild–moderate HI, but prevented the increase in brain damage over time. Additionally, rats treated with TH showed better long-term motor function. Altogether, our results bring more light to the understanding of pathophysiology following mild-moderate HI. We showed that, in the context of mild-moderate HI, TH failed to be significantly neuroprotective. However, animals treated with TH showed a significant improvement in motor, but not cognitive long-term function. These results are in line with what is observed in some cases where neonates with mild HIE are at risk of neurodevelopmental deficits in infancy or childhood. Whether TH should be used as a preventive treatment to reduce adverse outcomes in mild-HIE remains of active interest, and more research has to be carried out in order to address this question.

## 1. Introduction

Hypoxic ischemic encephalopathy (HIE) is one of the leading causes of morbidity and mortality in newborns, occurring in 2–3/1000 live births at term in high-income countries, with an even higher incidence in low- and middle-income countries [1,2]. HIE is caused by a lack of oxygen and/or blood flow to the brain before or during birth [3]. The underlying conditions leading to HIE are multifactorial, e.g., pre-eclampsia, placental abruption, prolapse of the umbilical cord, or respiratory arrest [1,4]. The most common long-term consequences are cerebral palsy, epilepsy, cognitive impairments, autism, and developmental delay [1,4]. Using different clinical categories (e.g., heart rate and neurological examination), HIE can be grouped in three degrees of severity: mild, moderate, and severe. Poor long-term outcomes are more generally described in moderate–severe HIE, but still vary from no (or only mild) effects to more severe and permanent disability [5]. In addition, neonates with mild HIE have been increasingly linked to adverse outcomes in the last years [6]. The pathological effects following HIE are not limited to a single event, and they can be described as an ongoing process divided into four different phases: (i) a primary energy failure directly after hypoxia-ischemia (HI) leading to neuronal death; (ii) a latent phase after restoration of oxygen and blood supply; (iii) a secondary energy failure phase associated mitochondrial dysfunction, delayed cells’ death, and inflammatory processes; and (iv) a tertiary injury process that goes on for months or years [3,4,7].

Therapeutic hypothermia (TH) is currently the standard treatment for newborns affected by moderate to severe HIE [2,8]. TH reduces mortality and improves functional outcome following moderate HIE in particular, but is only beneficial in approximately 50% of cases [4,9]. In severe HIE, as well as in other settings such as after perinatal infection, TH fails to be neuroprotective [10,11]. It also remains unclear whether TH is beneficial for babies affected by mild HIE [12], 1 in 4 of whom will develop neurodevelopmental disabilities [8]. It is thus essential to better understand the mechanisms underlying TH in the context of mild–moderate HI in order to develop alternative treatments, either as a single therapy or adjuvants in combination with TH. In ongoing preclinical and clinical studies, melatonin, erythropoietin, or allopurinol are some of the possible candidates [1,2,4]. Of note, the timing and temporal changes of brain injury in mild HIE are not well studied.

At birth, both human and rodent brains have not yet fully developed and matured [13]. Neurogenesis starts during the early embryonic stage and is still ongoing postnatally. A few neurogenic zones remain active even into adulthood [14]. One of these neurogenic niches is the subventricular zone (SVZ) adjacent to the lateral ventricles [15]. After HI, microglia are activated in the SVZ, and both pro- and anti-inflammatory cytokines, as well as neurotrophic genes, are upregulated, suggesting that HI will alter later neurogenesis [16,17]. However, current data concerning the effect of neonatal HI on neurogenesis are controversial; while some studies suggest that HI increases neurogenesis [16,18], others propose the opposite [19,20].

The aim of the current study was to substantiate the knowledge about the timing and the effects of mild–moderate HI in a clinically relevant rat model, not only with respect to neurogenesis, but also on structural and functional outcomes including cell-specific and trophic factor responses. Additionally, we analyzed the influence of TH on mild–moderate HI to help answer the question of whether TH might be beneficial for milder HI injuries.

## 2. Materials and Methods

### 2.1. Animals and Experimental Procedure

**Figure 1:**
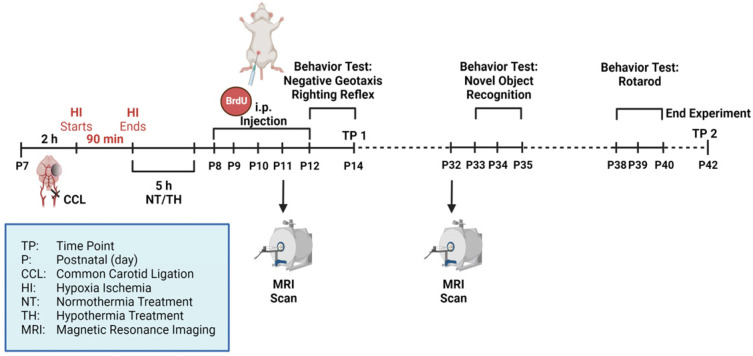
Experimental design: 7-day old Wistar rat pups (P7) were randomized into three groups: sham (n = 23), HI-NT (n = 23), and HI-TH (n = 21). In HI animals, ligation of the left common carotid artery was followed by hypoxia (8% O_2_, 36 °C) for 90 min and 5 h of NT (37 °C) or TH (32 °C) treatment. Rats were injected intraperitoneally (i.p.) with BrdU for 5 consecutive days at P8-P12. MRI scans were performed at P11 and P32. Behavior tests were performed between P12 and P40. Animals were either sacrificed at P14 (TP1) or P42 (TP2). Figure created with http://biorender.com (accessed on 9 May 2022).

All animal experiments were performed in accordance with the animal protection committee of the North Rhine-Westphalia State Environment Agency (LANUV), Germany. Reporting followed the ARRIVE guidelines. For all experiments, Wistar rat pups of both genders were used. Animals were housed at the central animal laboratory of the Deutsche Zentrum für Neurodegenerative Erkrankungen (DZNE) in Bonn, Germany, with a 12/12 h dark/light cycle at an environmental temperature of 21 °C and with food and water ad libitum. A total of 69 postnatal day (P) 7 Wistar rat pups were used (39 females and 30 males). Prior to the beginning of the experiment, animals were randomized by litter, sex, and weight to one of three different treatment groups: sham (n = 23), HI-NT (n = 23), and HI-TH (n = 21). Two animals were used as sentinels carrying temperature probes: n = 1 for NT and n = 1 for TH. These animals were not included in later analyses. The experimental timeline is outlined in Figure 1. After subcutaneous injection with 0.05 mg/kg bodyweight (BW) buprenorphine, animals from the HI group underwent ligation of the left common carotid artery under general anesthesia induced by 5% isoflurane. Sham animals were exposed to 5% isoflurane for 5 min without ligation. The HI group was then transferred to a temperature-controlled chamber and exposed to hypoxia at 8% O_2_ for 90 min at a rectal temperature (T_rectal_) of 36 °C. Sentinel pups carried a rectal probe (IT-21, Physitemp Instruments, Clifton, NJ, USA) connected to a servo-controlled cooling machine (CritiCool, MTRE, Yavne, Israel), which controlled a water-filled mat at the base of the chamber. Hypoxia was followed by 5 h of normothermia (NT) (T_rectal_ = 37 °C) or therapeutic hypothermia (TH) (T_rectal_ = 32 °C). All rats were injected with 100 mg/kg BW 5′-bromo-2′-deoxyuridine (BrdU) intraperitoneally (i.p.) for 5 consecutive days (P8-P12). BrdU is a thymidine analog that incorporates into the DNA of dividing cells and passes down in the following cell divisions [21]. Animals were either sacrificed at P14 (time point 1 = TP1) or P42 (TP2) by transcardial perfusion (animals used for histology) or decapitation (animals used for ELISA). For perfusion, phosphate-buffered solution (PBS) followed by 4% paraformaldehyde was used.

### 2.2. Histology/Immunohistochemistry and Definition of Brain Injury Severity

After sacrifice and transcardial perfusion, brains were post-fixed in 4% paraformaldehyde overnight at 4 °C. They were embedded in paraffin and cut into 10 µm coronal sections (Bregma, −0.3 mm and −3.8 mm). At both TP1 (P14) and TP2 (P42), hematoxylin and eosin (H&E) staining was performed for analysis of brain area loss, and immunohistochemistry staining was performed for neurons and microglia, as well as characterization of the neurogenesis of BrdU-positive cells. H&E staining was performed following standard protocol. The sizes of the ipsilateral (left) and the contralateral (right) hemisphere were measured using ImageJ (National Institutes of Health, USA) and area loss calculated with the following formula:

Area loss in

$$\%=\left(1-\frac{\mathrm{Area}\;\mathrm{ipsilateral}}{\mathrm{Area}\;\mathrm{contralateral}}\right)\times 100$$

The degree of brain injury was defined by the degree of area loss at TP1, as previously described [10,11,22,23,24]. In brief, mild HIE was defined as a median brain area loss <30%, moderate HIE as median brain area loss of 30–55%, and severe HIE as a median brain area loss >55%. Using this injury definition at P14, we have shown in multiple studies that TH is neuroprotective following moderate HIE, but failed to be protective following severe HIE [11,25,26,27,28,29,30].

For immunohistochemistry, double staining was performed. The slices were rehydrated using decreasing alcohol concentrations. Thereafter, antigen retrieval was performed by boiling in PBS for 7 min followed by permeabilization of the cell membrane with 0.1% Triton X-100 for 30 min at room temperature. The slices were blocked using 20% *v/v* (vol/vol) normal goat serum (Invitrogen, Darmstadt, Germany). The primary antibody (anti-Iba1, anti-NeuN) was incubated overnight at 4 °C followed by incubation of the corresponding secondary antibody for 1 h at room temperature (Table 1). Iba1 is a marker used for microglia [16] and NeuN is a marker used for mature neurons [31]. For BrdU-staining, the slices were incubated with 2M HCl for 30 min at room temperature, followed by incubation with the primary antibody overnight at 4 °C and with the corresponding secondary antibody for 1 h at room temperature (Table 1). Lastly, the slices were counterstained with 4,6-diamidino-2-phenylindole (DAPI) (Invitrogen, Germany). The slices were scanned with an AxioScan.Z1 (Carl Zeiss Microscopy GmbH, Oberkochen, Germany). For analysis, ZEN Blue 3.1 (Carl Zeiss Microscopy GmbH, Germany) and ImageJ were used. Three different regions of interest from the same coronal section (Bregma −3.8 mm) were analyzed: cortex (Ctx), hippocampus (Hipp), and lateral ventricle (LV). For the hippocampus and the lateral ventricle, the whole area in the slide was examined, while for the cortex, representative sections were analyzed. All positive cells (BrdU alone, co-localized Iba1–BrdU, or colocalized NeuN–BrdU) were counted. The number of cells was normalized to the size of the area analyzed to obtain the number of cells per mm^2^. Importantly, the degree of injury in some of the HI brains at TP2 was so high that immunohistochemistry could not be performed or analyzed. Therefore, at TP2, we only quantified those brains in which immunohistochemistry could be performed (Supplementary Materials Figure S1).

### 2.3. Magnetic Resonance Imaging (MRI)

MRI scans were performed at two time points within the same animals: P11 and P32. MRI was performed on an 11.7 Tesla (T) horizontal small-bore magnet (Biospec 117/16, Bruker, Billerica, MA, USA) using a rat brain receive only proton (1H) coil (Bruker Biospin). Anatomical images were acquired using a rapid acquisition relaxation enhancement (RARE) T2-weighted (T2-w) sequence (echo time (TE)  =  25 ms; repetition time (TR)  =  2.9 s; in-plane resolution 0.156 × 0.156 mm^2^). White matter integrity was assessed using an echo planar imaging diffusion tensor imaging (EPI DTI) sequence (TE/TR 30/3200 ms; b-value 650 s/mm^2^; 46 diffusion directions; 3 b0 images; in-plane resolution 0.15 × 0.15 mm^2^). Apparent diffusion coefficient (ADC) and fractional anisotropy (FA) were measured using ImageJ. ADC is the rate of the diffusion ability of water molecules in tissues [32,33]. FA also reflects the diffusivity of water molecules along fibers and considers different factors such as myelination, axonal integrity, and fiber diameter [34]. Tractography was performed using Diffusion Toolkit (Version 0.6.4.1, Athinoula A. Martinos Center for Biomedical Imaging, Charlestown, MA, USA) and TrackVis (Version 0.6.1, Athinoula A. Martinos Center for Biomedical Imaging, USA). Appropriate regions of interest (ROI) were drawn in the cortex and the hippocampus to perform the regional specific tractography. Area loss on MRI was calculated by measuring the area of both hemispheres for all slices using ImageJ and calculating the ratio as explained above. The severity of brain injury was defined at P11 in correspondence with the histological definition as explained above.

### 2.4. Behavior Testing

The following behavior tests were performed: Righting Reflex (P12–P14), Negative Geotaxis (P12–P14), Novel Object Recognition (P33–P35), and Rotarod (P38–P40).

The Righting Reflex is a test of motoric function and coordination [35]. Negative Geotaxis tests sensorimotor function [36]. Both were recorded on video and analyzed using Adobe Premiere (Adobe Systems Software Ireland Limited, Dublin, Ireland). Animals were trained for two days followed by testing on the third day. For the Righting Reflex Test, the pups were placed in a supine position, and the time it took them to change to a prone position was measured. For Negative Geotaxis, the pups were placed on a surface with an incline of 45° facing downwards, and the time it took them to face upwards with a 180° turn was measured. The cut-off time for both tests was 60 s.

The Novel Object Recognition (NOR) Test examines learning ability and memory [37]. NOR was performed over 3 days (P33–P35). Four 45 × 45 cm boxes with white walls and a black floor were used. On day 1 and day 2, each rat was put into an empty box for 10 min for habituation. On day 3, each rat was put into a box with two identical objects, placed in opposite corners of the box, for 5 min. After approximately 1 h, each rat was put into a box where the object was replaced by a novel object, for another 5 min. Each individual rat was placed into the same box each time to eliminate factors that may interfere with the results. The tests were recorded on video and analyzed using EthoVision XT 14 (Noldus Information Technology, Wageningen, The Netherlands). The ratio of the total time each rat spent exploring either of the two objects (*c*) to the time it spent exploring the novel object (*b*) was calculated to determine the percentage of time the rat spent exploring the novel object (*a*).

$$a=\frac{b}{c}\times 100$$

The Rotarod Test is another assessment of motor function [36], which was performed over 3 days (P38–P40). Rats were trained for two days; the third day was used for analysis. The rats were placed on a rotating barrel facing the wall with a starting speed of 4 rpm. The speed was then accelerated steadily to a maximum speed of 40 rpm. The time until animals fell off the barrel was measured and recorded. The cut-off time was 300 s = 5 min. Animals that deliberately stopped the test before reaching the maximum of their physical capability were excluded from the study.

### 2.5. ELISA

To determine the concentration of growth factors brain-derived neurotrophic factor (BDNF) and insulin-like growth factor 1 (IGF-1), ELISAs were performed. After sacrifice and decapitation, brains were separated into the two hemispheres and lysed with Hanks’ Balanced Salt Solution (gibco^TM^, Thermo Fisher Scientific, Waltham, MA, USA). For BDNF, Quantikine^®^ ELISA Total BDNF Immunoassay (R&D Systems, Inc., Minneapolis, MN, USA) was performed following the manufacturer’s instructions with a dilution of 1:100 for brain lysate. For the analysis of IGF-1, Quantikine^®^ ELISA Mouse/Rat IGF-I/IGF-1 Immunoassay (R&D Systems, Inc., Minneapolis, MN, USA) was performed following the manufacturer’s instructions with a dilution of 1:5 for the brain lysate.

### 2.6. Statistical Analysis

GraphPad Prism 9.1.2 (GraphPad Software, San Diego, CA, USA) was used to analyze and plot the data. For the histology, behavioral testing, and ELISA results, a Mann–Whitney test was performed. For MRI, an unpaired t-test was performed. All data are presented as the median with interquartile range. *p*-values < 0.05 were considered statistically significant.

## 3. Results

### 3.1. Neonatal Hypoxia-Ischemia Leads to Significant Morphological and Structural Brain Damage

As expected, compared with the sham group, a significant area loss was noted following HI. At TP1, the ipsilateral hemisphere was on average 20% smaller than the contralateral hemisphere, corresponding to mild HIE in our animal model. However, over time, the extent of the damage increased greatly, leading to a size difference of over 50% on average.

**Figure 2:**
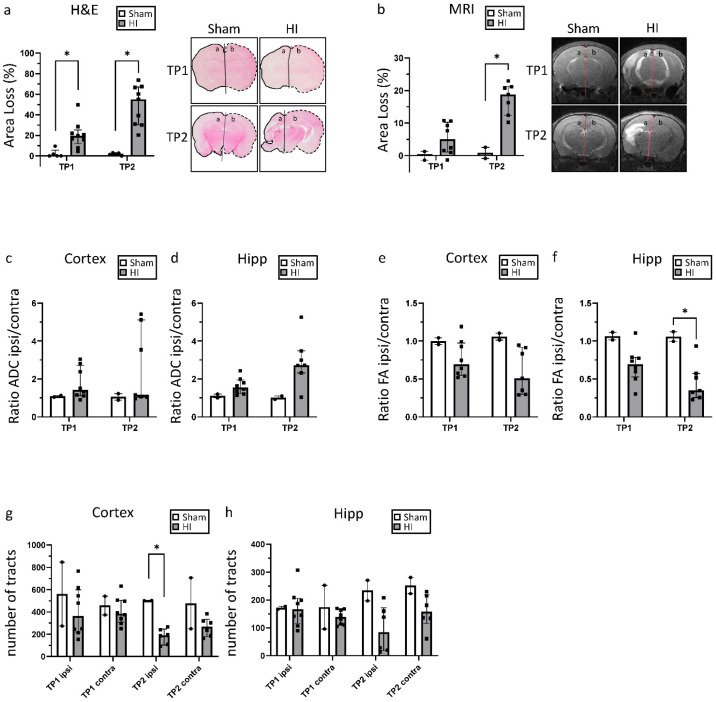
Morphological and structural brain damage: (**a**) H&E staining and area loss of the ipsilateral hemisphere (marked a) compared with the contralateral hemisphere (marked b); (**b**) area loss of the ipsilateral hemisphere (marked a) compared with the contralateral hemisphere (marked b) measured in T2-weighted MRI images; (**c**,**d**) ratio of ADC values from the ipsilateral and the contralateral hemisphere in the cortex (**c**) and the hippocampus (**d**); (**e**,**f**) ratio of FA values from the ipsilateral and the contralateral hemisphere in the cortex (**e**) and the hippocampus (**f**); (**g**,**h**) number of tracts measured in tractography for the cortex (**g**) and the hippocampus (**h**). For (**a**), TP1 = P14 and TP2 = P42; for (**b–h**), TP1 = P11 and TP2 = P32. ADC = apparent diffusion coefficient. FA = fractional anisotropy. HI = hypoxia-ischemia. TP = time point. * *p* ≤ 0.05. Group sizes: (**a**) sham n = 5, HI n = 9; (**b**–**f**) sham n = 2, HI TP1 n = 8, TP2 n = 7; (**g**,**h**) sham n = 2, HI TP1 n = 8, TP2 n = 6.

Similar results were seen from the analysis of T2-weighted MRI images at P11 and P32 (Figure 2b), where the same animals could be assessed at both time points. Brain damage increased over time, rising from approximately 5% area loss at P11 to 19% loss at P32. Considering each animal individually, area loss increased by between 5 and 21 percentage points (Supplementary Materials Figure S2) between the two time points. Lesion size as a percentage of the whole brain, which mainly reflects the extent of brain edema, is shown in Supplementary Materials Figure S3.

ADC and FA were measured to assess the extent of diffusion restrictions in the cortex and the hippocampus (Figure 2c–f). To normalize the values for each animal, the ratio between ipsilateral and contralateral was calculated. In the HI group, ADC values increased in the ipsilateral compared with the contralateral hemisphere at both timepoints. This imbalance also increased between the two time points; especially in the hippocampus. Similar results were found for the FA. In the HI group, FA values were lower in the ipsilateral than in the contralateral hemisphere at both time points.

To assess the extent of structural integrity of the major white matter, tractography was performed for the cortex and hippocampus. A decrease in tracts in the HI group compared with Sham was found for both time points and regions. Interestingly, these changes were found on both the ipsilateral and contralateral sides. A particularly strong decrease could be noted for the second time point for both the cortex and the hippocampus.

### 3.2. Effect of HI on Functional Outcome

**Figure 3:**
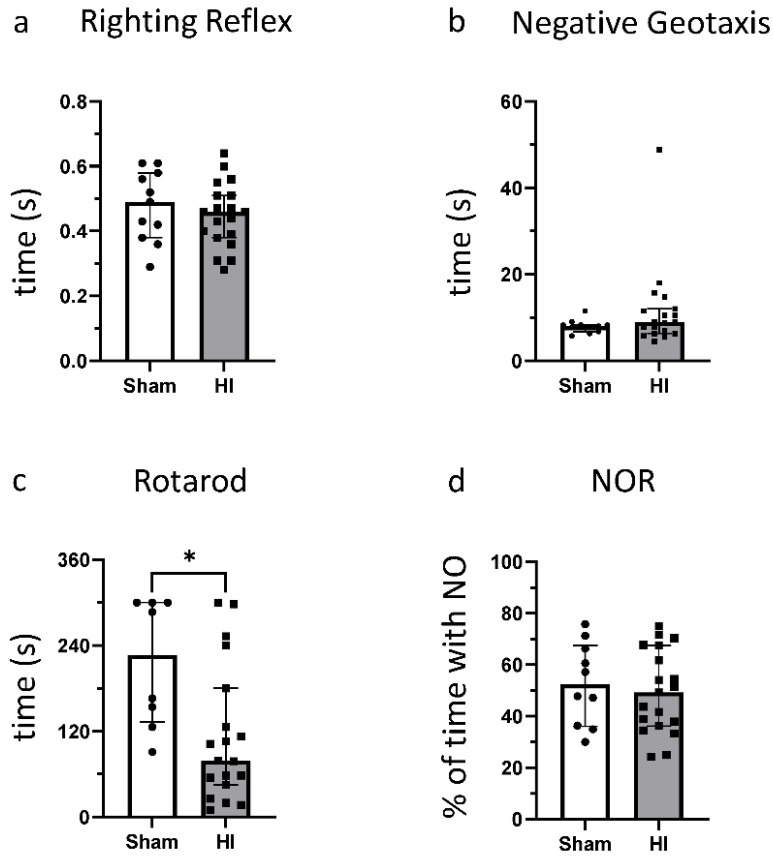
Effects of HI on behavioral outcomes: (**a**) Righting Reflex; (**b**) Negative Geotaxis; (**c**) Rotarod; and (**d**) Novel Object Recognition. * *p* ≤ 0.05. Group sizes: (**a**,**b**,**d**) sham n = 11, HI n = 19; (**c**) sham n = 8, HI n = 19.

At P14, neither the Righting Reflex nor the Negative Geotaxis showed any difference between the injured animals and sham animals (Figure 3a,b). HI animals also did not differ from sham animals in the NOR test (Figure 3d). In the Rotarod Test, a significant decrease in the time the animals could stay on the rotating barrel was noted (Figure 3c). These data show that, in approximately 5-week-old rats, mild–moderate HI insult results in motor, but not cognitive impairment compared with sham control.

### 3.3. Effect of HI on Neurogenesis

**Figure 4:**
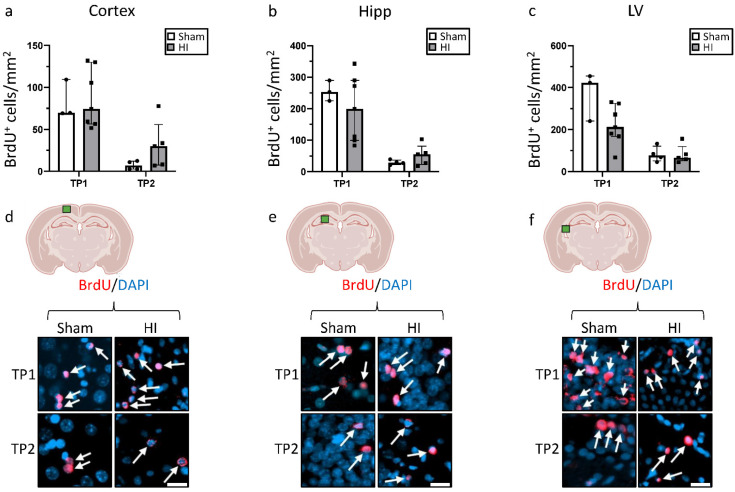
Effect of HI on cell proliferation in different brain regions: Number of BrdU-positive cells in (**a**) the cortex; (**b**) the hippocampus, and (**c**) the lateral ventricle; (**d**–**f**) immunostaining of the cortical and hippocampal area, respectively, showing BrdU-positive cells. TP1 = P14. TP2 = P42. Group sizes: TP1 sham n = 3, HI n = 8; TP2 sham n = 3, HI n = 4. TP1 vs. TP2 *p* < 0.05. Scale bar = 20 µm.

At TP1, we observed a non-significant decrease in the number of BrdU^+^ cells in HI in the hippocampus and lateral ventricle, whereas we did not observe changes in the cortex (Figure 4). By comparison, at TP2, the number of BrdU^+^ cells was slightly higher, but not significantly, for HI animals in the cortex and the hippocampus, with no difference in the lateral ventricle. In general, the number of BrdU^+^ cells was significantly higher at TP1 than at TP2 for both groups (*p* < 0.05), and in all three regions.

**Figure 5:**
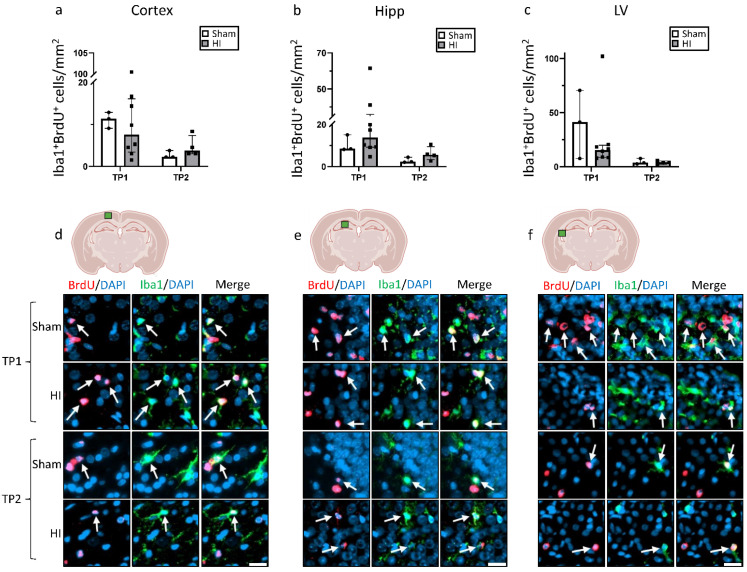
New proliferating cells after HI. Quantitative analysis of microglia cells (Iba1+) that co-localized with BrdU+ cells in (**a**) the cortex, (**b**) the hippocampus, and (**c**) the lateral ventricle; (**d**–**f**) representative immunostaining of Iba1+/BrdU+ cells in the corresponding brain regions. TP1 = P14. TP2 = P42. Group sizes: TP1 sham n = 3, HI n = 8; TP2 sham n = 4, HI n = 4. Scale bar = 20 µm.

**Figure 6:**
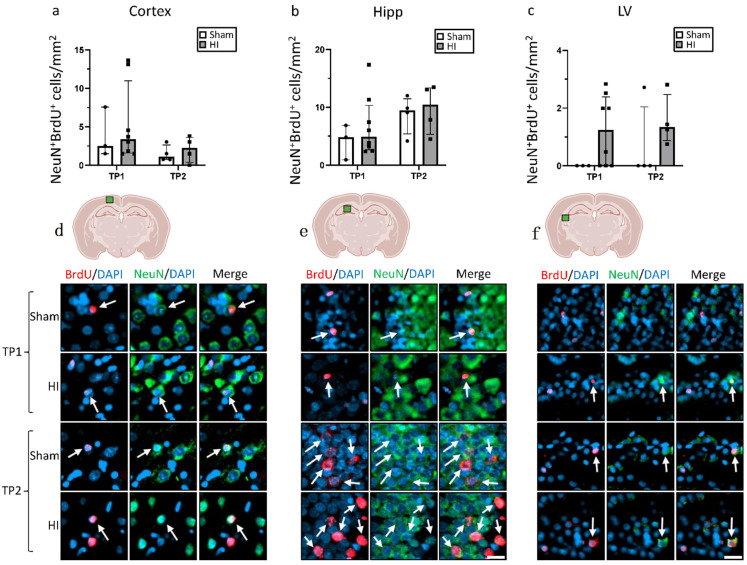
New proliferating cells after HI. Quantitative analysis of NeuN+ neurons that co-localized with BrdU+ cells in (**a**) the cortex, (**b**) the hippocampus, and (**c**) the lateral ventricle; (**d**–**f**) representative immunostaining showing NeuN+/BrdU+ cells in the corresponding brain regions. TP1 = P14. TP2 = P42. Group sizes: TP1 sham n = 3; HI n = 8, TP2 sham n = 4, HI n = 4. Scale bar = 20 µm.

To further investigate the nature of the new cells, double staining for BrdU in combination with either Iba1 or NeuN was performed (Figure 5 and Figure 6). In the cortex, the number of Iba1^+^ + BrdU^+^ cells was non-significantly increased in sham animals at TP1, and the opposite was seen at TP2 (Figure 5a). In the hippocampus, a non-significant increase in Iba1^+^ + BrdU^+^ cells was observed in the HI group at both TPs, especially for TP1 (Figure 5b). In the lateral ventricle, the number of Iba1^+^ + BrdU^+^ cells was lower for the HI-NT animals at TP1 and very similar at TP2.

The number of NeuN^+^ + BrdU^+^ cells was generally very low for both groups at both TPs in all regions, especially in the lateral ventricle (Figure 6).

### 3.4. HI Leads to a Significant Reduction in Fundamental Growth Factors

**Figure 7:**
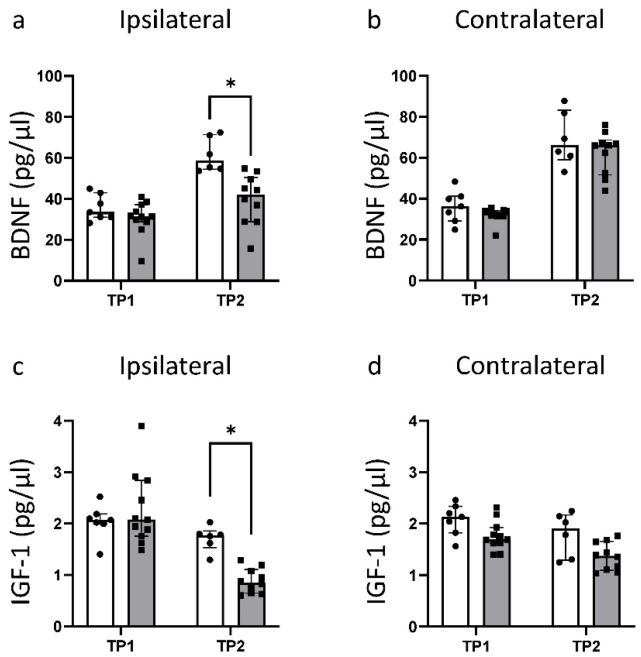
Measurement of the expression level of growth factors BDNF and IGF-1 in brain lysates: (**a**) concentration of BDNF in pg/µL in the ipsilateral hemisphere; (**b**) concentration of BDNF in pg/µL in the contralateral hemisphere; (**c**) concentration of IGF-1 in pg/µL in the ipsilateral hemisphere; (**d**) concentration of IGF-1 in pg/µL in the contralateral hemisphere. TP1 = P14. TP2 = P42. * *p* ≤ 0.05. Group sizes: TP1 sham n = 7, HI n = 11; TP2 sham n = 6, HI n = 10.

In healthy animals, the concentration of BDNF was similar in both hemispheres, and increased significantly between TP1 and TP2 (Figure 7a,b). While the ipsilateral BDNF values were very similar between sham and HI at TP1, a reduction in BDNF could be noted at TP2 in the HI group. Here, HI led to a significant reduction to almost 70% of the sham level (Figure 7a).

The IGF-1 concentration decreased for both groups and in both hemispheres between TP1 and TP2 (Figure 7c,d). This decrease over time was more pronounced for HI animals. While the IGF-1 concentration on the ipsilateral hemisphere for HI animals was very similar to the sham level at TP1, it showed a significant reduction to less than 50% of the sham value at TP2.

### 3.5. Effects of Hypothermia Treatment

**Figure 8:**
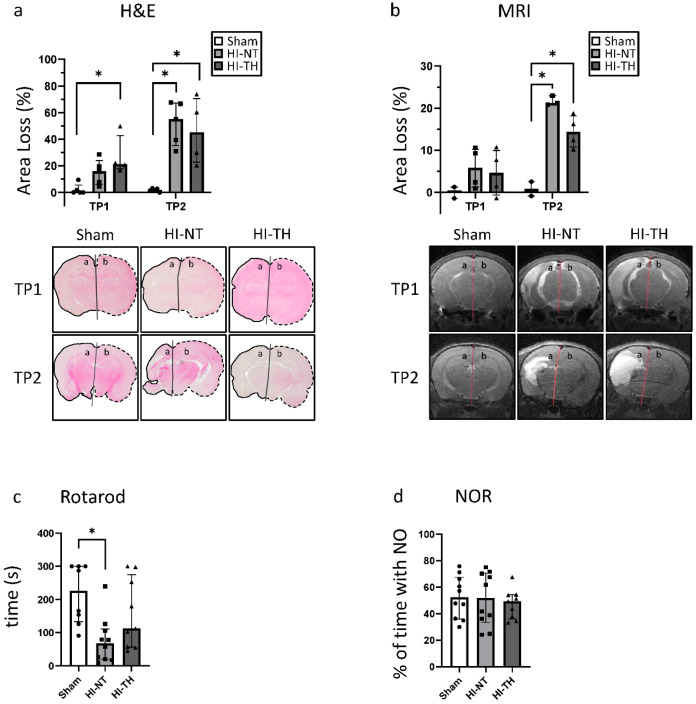
Effects of hypothermia treatment after HI: (**a**) area loss of the ipsilateral hemisphere (marked a) compared with the contralateral hemisphere (marked b) measured with H&E staining; (**b**) area loss of the ipsilateral hemisphere (marked with a) compared with the contralateral hemisphere (marked with b) measured in T2-weighted MRI images; (**c**) Rotarod Test; (**d**) Novel Object Recognition Test. For (**a**), TP1 = P14 and TP2 = P42; for (**b**), TP1 = P11 and TP2 = P32. * *p* ≤ 0.05. Group sizes: (**a**) sham n = 5, HI-NT n = 5, HI-TH n = 4; (**b**) sham n = 2, HI-NT TP1 n = 4, TP2 n = 3, HI-TH n = 4; (**c**) sham n = 8, HI-NT n = 10, HI-TH n = 9; (**d**) sham n = 11, HI-NT n = 10, HI-TH n = 9.

At TP1, no significant neuroprotective effects of TH were observed with either histology or MRI (Figure 8a,b). At TP2, the measured area loss was smaller for animals treated with HI-TH compared with HI-NT, especially in the MRI. This signifies that TH may prevent the aggravation of the existing brain damage over time following mild–moderate HIE. While the area loss increased by 11.54 percentage points in HI-NT animals, this number was reduced to 9.81 in HI-TH animals (Supplementary Materials Figure S2). While the cognitive function of the rats was not affected by TH (Figure 8d), a significant improvement in motor function could be observed in the Rotarod Test (Figure 8c).

## 4. Discussion

HIE is a major contributor to neonatal mortality and remains a big challenge for clinicians [4]. In this project, the impact of mild–moderate HI in a rat model of HIE was examined. We demonstrate progression of the pathological changes in a mild–moderate insult using MRI, histological, and functional outcomes. Mild–moderate HI leads to a progressive loss of brain tissue and impairment of motor function over time. We also examined the effectiveness of TH in the context of mild–moderate HI and showed that TH improves motor outcomes and provides a small benefit in slowing the progression of brain tissue loss.

As the injury after HI is an ongoing process that can last months and even years [4,7], it is unsurprising that the loss of the damaged brain hemisphere increases after the initial insult. Similarly, it is well described that the severity of some cases of mild-HIE can evolve over time to become more severe at 48 to 72 h. This signifies that brain tissue can still become injured long after the initial insult, and the damaged brain hemisphere cannot develop normally. However, once more than about six hours have passed since the injury, TH is no longer beneficial [6,38,39,40,41]. To substantiate these findings in our model, we assessed area loss using two different methods: histology and T2-weighted MRI scans. MRI including DTI is the gold standard for diagnosing HIE [33]. Neonatal MRIs are also of great prognostic value as they are a good predictor of the functional outcome [33,42,43]. Three different regions and patterns of injury can be predominantly distinguished: (1) basal ganglia and thalamus; (2) white matter; and (3) global injury [33]. However, when using a small animal model, the exact areas affected by the injury cannot be directly translated. Therefore, in this study, we only analyzed the cortex and the hippocampus. In addition to T2-weighted images, we also analyzed ADC and FA using DTI, which are both markers for the diffusion of water molecules [32,33,34]. In a similar study, a decrease in ADC was seen at very early time points after HI, with a later increase after 3 days [44]. This increase proved to still be pronounced and could be explained by compromised tissue integrity and cell death [44,45,46]. The observed decrease in FA reflects a destruction of fibers in the brain [47]. In our study, increases in ADC and decreases in FA become more pronounced from TP1 to TP2, further supporting the area loss that shows that the injury worsens over time.

Considering the extent of damage resulting from HI shown in the present study, a clear decrease in tracts measured by tractography is to be expected. This decrease is especially distinct at TP2 in both the cortex and the hippocampus. Interestingly, the number of tracts in sham animals remained approximately the same over time or even increased, whereas it decreased for HI animals. This shows once more that the impact of the injury increases over time, resulting in ongoing impaired brain development. It would be reasonable for the contralateral side to compensate at least partly for the ipsilateral hemisphere, as has been observed after ischemic stroke in adults [48]; interestingly, however, a decrease in tracts was also seen in the contralateral hemisphere, especially at TP2. This implies that, in this model, HI also impairs the development of the “unaffected” contralateral side.

Cell death is a crucial part of HIE pathophysiology, and the process of formation of functional mature neurons through neurogenesis is important after an injury, but neurogenesis is still not well described in HIE. While other studies have showed an increase in BrdU^+^ cells after HI [7,13], we could not find a significant increase in BrdU^+^ cells in the cortex, the hippocampus, or the lateral ventricle in our mild–moderate model. Most of the data available on neurogenesis in HI animal models focused on the SVZ [7,13,31], but in the present study, we focused on broader parts of the brain. This could imply that, while neurogenesis in the SVZ is stimulated after HI, the same is not the case for other areas of the brain, or that new cells do not migrate across the injured brain at the time points analyzed. It can also be seen that the number of BrdU^+^ cells decreases significantly between the two time points for both groups. This could imply that, even if HI does stimulate neurogenesis, new cells may not survive in the long term at this time of development, perhaps because of HI negatively affecting the environment necessary for neuronal proliferation and maturation. For example, an increase in oxidative stress occurs after HI [4], which has been associated with a reduction in neurogenesis [49]. The reduction in growth factors as shown in this study is another aspect that may contribute to the lack of a proper environment for neurogenesis. By counting the NeuN/BrdU double-positive cells, we also showed that only a minor fraction of the new cells develop into neurons. However, the evidence currently published concerning NeuN/BrdU double-positive cells after HI is controversial—a decrease, an increase, and no change have all been previously shown to occur [50,51,52,53].

As microglia play an important role during neurogenesis, Iba1/BrdU double-positive cells were also analyzed. In both the cortex and the hippocampus, we showed that the number of microglia was higher after HI, especially at TP1. As microglia have both positive and negative effects on the development of the brain based on phenotype and activation status [4,22,24,54], further research is needed to understand the impact of these cells in mild–moderate HI. However, assessment of new cells is still relatively limited in our model, particularly at TP2, because there is an increase in the area affected in the brain, and because of the variability in injury between animals in this model. Because of this, further studies need to be performed concerning region-specific neurogenesis over time using larger group sizes.

The development and maturation of the brain is controlled by different growth factors [14,55,56]. In the developing brain, insulin-like growth factor 1 (IGF-1) is important for neuronal and glial proliferation and differentiation, as well as for synapse formation and synaptic plasticity [15,55,57,58]. Brain-derived neurotrophic factor (BDNF) supports neurogenesis, neuronal survival, synaptic plasticity, and dendrite growth and is important for recovery after brain damage [56,58,59,60]. In addition, BDNF expression is upregulated by IGF-1 [61]. IGF-1 expression in the brain is at its highest prenatally and decreases greatly after birth [55,57]. In concordance to this, we detected higher IGF-1 levels at TP1 than at TP2. In a similar rat model, an increase in BDNF mRNA levels was detected for very early time points (6–24 h after injury), while a decrease in BDNF protein expression was measured [56]. In our study, we showed that this decrease in protein levels is still present at later time points. In a previous study, an increase in BDNF protein levels in the forebrain at P42 in a mouse neonatal HIE model was described [62]. This discrepancy may be explained by the fact that we used whole brain lysates and BDNF expression may vary for different brain regions. Alternatively, there may be differences between mouse and rat brains with respect to the model used or response to injury. A reduction in both IGF-1 and BDNF caused by HI, as we demonstrated in our model, will likely contribute to the evolving brain damage by reducing in the development of new neuronal cells. We also showed that there is a difference in the expression of both IGF-1 and BDNF between the two hemispheres, especially at TP2, which may be a sign of a disrupted interhemispheric connection [62].

To assess functional impairment following HI, multiple behavior tests were performed. The Righting Reflex test and the Negative Geotaxis test showed that, a few days after the mild-moderate HI insult, basic reflexes were not affected. In contrast to that, in a similar model, rats treated with HI were significantly slower in the Negative Geotaxis test (but not in the Righting Reflex test) [36]. This could be because of the high variability of the model or the milder injury in our model, as well as the variability in behavior tests in general. For Negative Geotaxis in particular, a few animals in the HI group showed very slow response times, reinforcing once more the high variability in injury and outcome even in a milder version of this model. At approximately 5 weeks, equivalent to early adolescence in rats [63], cognitive function was also similar for animals with and without brain damage; however, a clear impairment of motor function was seen. It has to be noted that the results of the Novel Object Recognition Test may not be conclusive, as even the sham animals did not show a preference for exploring the novel object as opposed to the known object. It may be possible that the animals were not mature enough yet at 5 weeks, as in other studies, the rats were older when performing the NOR [63,64,65,66].

Similar to our work, other studies have found a significant deterioration in the Rotarod Test after HI [36], and these findings are consistent with the symptoms of babies affected by HIE. MRI studies suggest that HIE especially affects brain areas important for tone and movement [7]. While motor impairment is very common after HIE, cognitive impairment can also depend on the severity and the affected brain regions, even in the absence of motor damage [7,43]. Studies have also revealed that cognitive impairment can manifest during adolescence after neonatal injury, and may not yet be manifested during early childhood [43]. After mild HIE, cognitive impairment but normal language skills and motor function have been reported, but often the long-term outcome of babies affected by mild HIE is similar to after moderate HIE [67,68]. As we only analyzed the short-term outcome, and the animals were still very young, long-term behavior tests should be performed to assess the cognitive and motor development of the rats.

As TH is the current standard treatment for babies affected by moderate–severe HIE, but knowledge is lacking concerning TH in the context of mild HIE, we also analyzed the effect of TH in our model. Here, we found an amelioration of motor deficits after TH treatment compared with HI-NT, and no change in cognitive function. Similar results were also shown in previous studies at later time points (P50-P70) using NOR as a test for cognitive outcome and the Beam Walk test for motor outcome [65]. Likewise, in a mouse HI model, TH also protected against motor but not memory deficits [62]. Another important aspect is the morphological extent of the brain damage. Similar to other studies [65], we were able to show that, while the area loss is very similar for HI-NT and HI-TH at early time points, TH seems to prevent the worsening of the damage over time. Particular emphasis has to be placed on the potential negative aspects of TH. In severe HIE, TH is considered safe, but as mentioned several times, knowledge is lacking in the context of mild HIE [8]. These babies are in a much better health condition, and TH may lead to significant shivering and stress, as well as additional medical interventions such as sedatives that are known to have potential neurotoxic effects, and that could thus compromise any benefits of treatment [8,69].

Understanding how the brain recovers after an insult is crucial to improve treatment and to develop new or alternative treatments. It is clear that, to some extent, the brain is able to develop and support new cells to compensate for an injury this early age. However, we observed a high variability in the extent of brain damage resulting from HI as well as in the number of new proliferating cells in the affected brains between different subjects. The same problem occurs in human neonates affected by HIE [70]. While, normally, in the field, only histology is used to assess area loss in animal models, we additionally used MRI. This enhances the conclusions drawn from histology, but also gives further information about the connectivity of the brain. We also showed that, even though treatment with TH shows a high variability, it protects against an exacerbation of brain damage in some animals.

This study helps to acquire a deeper understanding of how the brain responds after a mild–moderate HI injury, especially concerning the proliferation and maturation of new cells, and how those change over time. Altogether, our results bring a greater understanding of pathophysiology following mild–moderate HI, particularly in the context of TH following mild–moderate HI. Keeping in consideration the balance between the benefits and the risks of TH in mild–moderate HIE, the positive effects of TH do not necessarily outweigh the negative effects observed in clinical settings. This draws the conclusion that, while clinical trials of TH for mild HIE are sorely needed, TH should only routinely be administered in moderate–severe cases of HIE. Alternative treatment options need to be developed with fewer negative side effects for those mild–moderate HIE cases, and this is particularly true for those needing delayed treatment or in other settings where TH is not neuroprotective.

## Figures and Tables

**Table 1:** Antibody list used for immunohistochemistry.

**Primary Antibodies**	**Company**	**Host**	**Catalogue No.**	Dilution *
anti-BrdU	Developmental Studies Hybridoma Bank, Iowa City, IA, USA	mouse	AB_2314035	1:100
anti-Iba1	Wako, Neuss, Germany	rabbit	AB_839504	1:200
anti-NeuN	Cell Signaling Technology, Danvers, MA, USA	rabbit	#24307	1:100
**Secondary Antibodies**	**Company**	**Host**	**Catalogue No.**	Dilution *
Goat Anti-Mouse Alexa Fluor^TM^ 594	Invitrogen, Waltham, MA, USA	goat	AB_2534091	1:300
Goat Anti-Rabbit Alexa Fluor^TM^ 488	Invitrogen, Waltham, MA, USA	goat	AB_143165	1:300

* All dilutions were performed using 0.7% Carrageenan solution with 0.02% NaN_3_ solution in PBS 1×.

## Data Availability

Not applicable.

## Supplementary Materials

The following supporting information can be downloaded at https://www.mdpi.com/article/10.3390/life12081164/s1, Figure S1: (a–g) Effect of TH on proliferation of new cells, Figure S2: (a,b) Area loss over time, Figure S3: (a,b) Brain lesion volume.
