# Supplementary material for: Impact of Hypoxia-Ischemia on Neurogenesis and Structural and Functional Outcomes in a Mild–Moderate Neonatal Hypoxia-Ischemia Brain Injury Model
Source: Life (Basel). 2022 Jul 30;12(8):1164. doi: 10.3390/life12081164 (PMC9410039; doi:10.3390/life12081164)

Supplementary Material Figure S1:(a-g) Effect of TH on proliferation of new cells. * $p \leq 0.05$

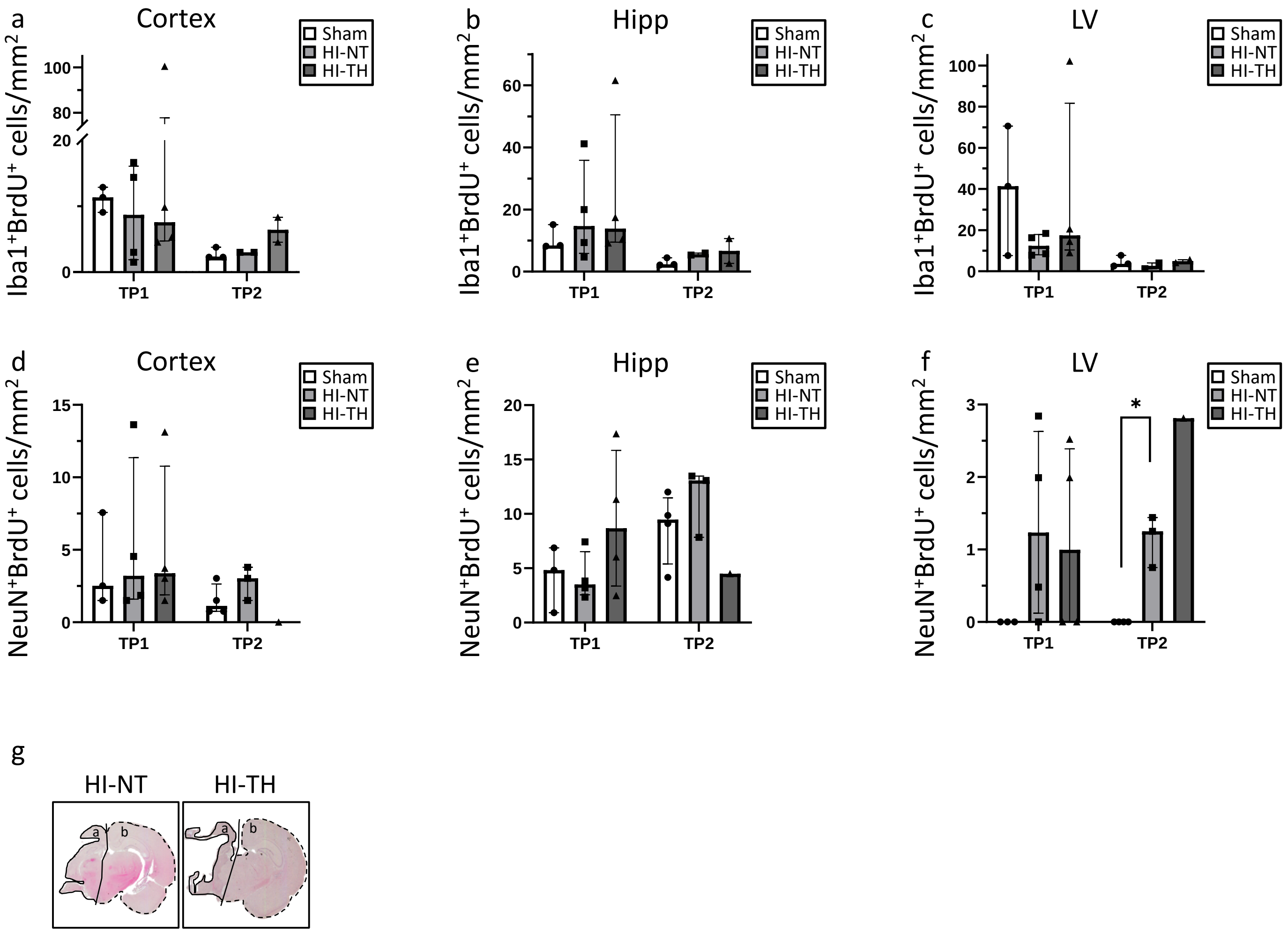

Supplementary Material Figure S2:(a,b) Area loss over time, * p ≤ 0.05

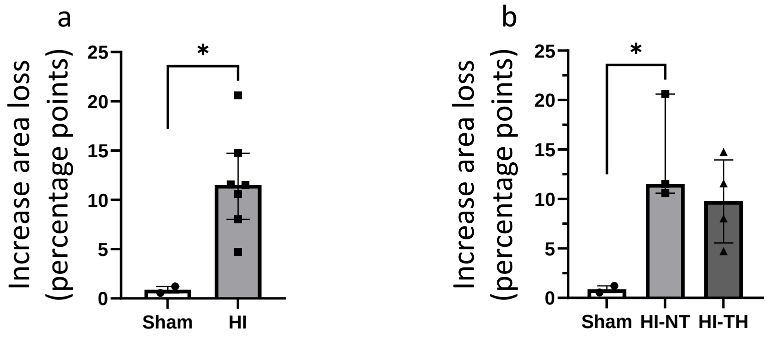

Supplementary Material Figure S3:(a,b) Brain lesion volume * p ≤ 0.05

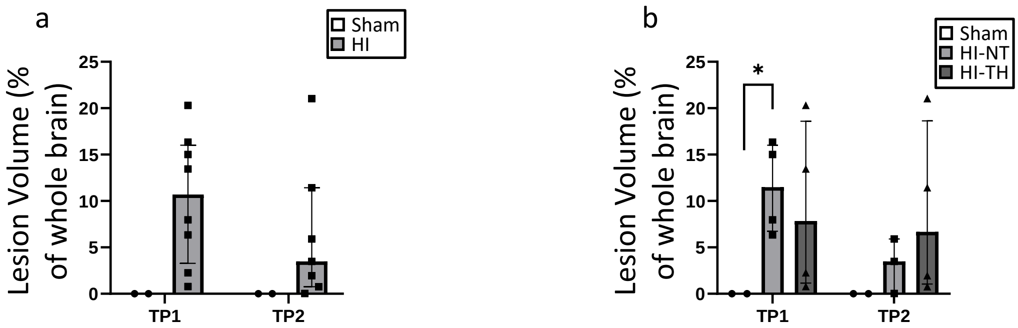

Supplement: Supplementary file 1 [file life-12-01164-s001.zip › life-1821903-supplementary.pdf]
